# The Code of Ethics of the New York State Medical Society and the American Medical Association

**Published:** 1882-05

**Authors:** 


### EDITORIAL DEPARTMENT.

The Code of Ethics of the New York State Medical Society and the American Medical Association.—We have hitherto refrained from commenting on the important modifications made in the code of ethics at the last meeting of the New York State Medical Society. We have done so principally because questions of ethics, even of medical ethics, must eventually be settled by each one for himself, according to the dictates of his conscience and his understanding of the duties he owes to his fellow-practitioners and the public. As a general guide to which to conform, a written code is useful, perhaps necessary; but it is questionable whether a strict construction of the provisions of an arbitrary ethical rule is calculated to secure the best results to all concerned. In nearly all relations of life the physician needs to consider the effect of his actions upon the public, his patrons, as well as upon his fellow-practitioners. Toward each other our duties are like unto those of honorable business men toward one another. We must be honorable in all our dealings with the whole world—no self-respecting man can accept any other rule of action. We may not underbid each other in fees; we may not depreciate each other's qualifications or attainments; we shall always shield—so far as conscience allows—those who may have been unfortunate. These propositions all will accept and observe, whether they form parts of a written code of ethics or not. They are not medical ethics, but fundamental principles of morality.

But we have, in the code of ethics of the American Medical Association, a definite rule governing consultations. According to this rule no consultation shall be held with any one "whose education is based on an exclusive dogma to the rejection of the accumulated experience of the profession," etc. This, of course, excludes homœopathists from consultations. Now, at the recent meeting of the State Medical Society this rule was modified so as to read as follows:

> "Members of the Medical Society of the State of New York, and of the medical societies in affiliation therewith, may meet, in consultation, legally qualified practitioners of medicine. *Emergencies may occur in which all restrictions should in the judgment of the practitioner yield to the demands of humanity.*"

Inasmuch as by the laws of New York, and, we presume, most of the other States, homœopathists, hydropathists, eclectics, or any others who possess a diploma from a chartered college, are recognized as "legally qualified" practitioners, the restrictions of the national code are abrogated by the section above quoted. This has raised a perfect howl of indignation from medical journals in various parts of the country. Except in this city, we have been able to find only two journals which have expressed approval in any way with modications in the code noted above. All the rest which have referred to the matter have done so in terms, more or less strong, of disapproval. Some of these journals have gone quite beyond the limits of decency in their comments, and have imputed the most unworthy motives to the framers and supporters of the new code. It is of no consequence that the gentlemen who were most active in the adoption of this modified ethical guide are among the most eminent in the profession, not only in New York, but in the country. They are accused of a covert desire to secure a portion of the consulting practice of the homœopathists, of an anxiety to increase their income by disreputable means, and to crown all they are called "specialists" as if it were a term of reproach, and as if a "specialist" were a sort of excrescence on the medical body politic who had no right to existence except by the special grace of the general practitioner. We need hardly call attention to the fact that a large portion of the scientific and practical medical work in this country is due to these at present much abused members of the profession. Let the able editors who are so strenuously opposed to the New York code look through the files of their own journals or their exchanges and note the amount of toll paid to general medical literature by these same New York specialists.

But the question which every one has in the end to determine for himself is: Is it right? To this complexion it must come at last. Shall any physician, being called upon in a case in which humanity demands aid—which he alone may at the time be able to give—refuse it until a practitioner having charge of the case, who may be practising according to a system deemed false by the first, be summarily dismissed? Has he any moral right to refuse his assistance in such a case on the ground that the code of ethics does not permit him to yield to the demands of humanity, when complicated by the presence of a homœopath or eclectic? Unquestionably not. He need not turn homœopathist, hydropathist or eclectic for the time being. The very fact that he has been called in consultation is evidence that the attending physician doubts the sufficiency of his own methods to deal with the case in hand.

Aside, however, from the purely ethical question involved, a question of medical polity has also arisen in this connection. It is confidently asserted by those who so clamorously assume to be the conservators of, the medical honor of the nation, that the delegation from the New York State Society will not be admitted at the next annual meeting of the American Medical Association. We may be permitted to doubt whether any such suicidal action will be taken. On looking through the volume of transactions of the last annual meeting of the association, we find that one fourth of all the papers read at that meeting were contributed by New York members and delegates. It would seem that any such disciplining of the New York Society as is threatened by the journals throughout the country would have a bad effect upon the prosperity of the National Association. It is absurd to suppose that breaking off official relations with the most eminent medical men in the country would in any degree limit the influence which, as teachers, they exert upon medical opinion throughout the country.

